# Wastewater Characterization: Chemical Oxygen Demand or Total Organic Carbon Content Measurement?

**DOI:** 10.3390/molecules29020405

**Published:** 2024-01-14

**Authors:** László Wojnárovits, Renáta Homlok, Krisztina Kovács, Anna Tegze, Ezsébet Takács

**Affiliations:** HUN-REN Centre for Energy Research, Konkoly-Thege M. út 29-33, 1121 Budapest, Hungary; wojnarovits.laszlo@ek-cer.hu (L.W.); homlok.renata@ek.hun-ren.hu (R.H.); kovacs.krisztina@ek.hun-ren.hu (K.K.); tegze.anna@ek.hun-ren.hu (A.T.)

**Keywords:** pharmaceuticals, advanced oxidation processes, chemical oxygen demand, total organic carbon, gamma radiolysis

## Abstract

The long time (2 h) required for measurement, expensive chemicals (Ag_2_SO_4_), and toxic reagents (K_2_Cr_2_O_7_, HgSO_4_) limit the application of the standard method for measuring the oxygen equivalent of organic content in wastewater (chemical oxygen demand, COD). In recent years, the COD has increasingly been replaced by the total organic carbon (TOC) parameter. Since the limit values of the pollution levels are usually given in terms of the COD, efforts are being made to find the correlation between these parameters. Several papers have published correlation analyses of COD and TOC for industrial and municipal wastewater, but the relationship has not been discussed for individual chemicals. Here, this relationship was investigated using 70 contaminants (laboratory chemicals, pharmaceuticals, and pesticides). The calculated COD values, in most cases, agreed, within ~10%, with the experimental ones; for tetracyclines and some chloroaromatic molecules, the measured values were 20–50% lower than the calculated values. The COD/TOC ratios were between 2 and 3: for macrolides, they were ~3; for fluoroquinolones and tetracyclines, they were ~2. The molecular structure dependence of the ratio necessitates the establishing of the correlation on an individual basis. In advanced oxidation processes (AOPs), the ratio changes during degradation, limiting the application of TOC instead of COD.

## 1. Introduction

The organic carbon in water/wastewater originates from a variety of organic compounds in various oxidation states. Some of these carbon compounds can be oxidized through chemical or biological processes, and the routinely applied chemical oxygen demand (COD_meas_) and biochemical oxygen demand (BOD) analyses are typically used to characterize these fractions [1,2]. COD_meas_ measures the oxygen equivalent (mg O_2_ dm^−3^) of the organic matter content of the sample, susceptible to oxidation by strong chemical oxidants (potassium permanganate or potassium dichromate) [3]. Potassium dichromate is mainly used for assessing the water quality in moderately or heavily contaminated water bodies (like in wastewater treatment plants), while potassium permanganate is often used as an oxidant for relatively clean water (like surface water). For the BOD, the digestion is carried out using a mixed microorganism population collected from the purification system of a wastewater treatment plant [4].

The classical COD_meas_ analytical technique using dichromate is based on refluxing a 10 mL sample for 2 h in a sulfuric acidic medium containing potassium dichromate (K_2_Cr_2_O_7_) in the presence of a silver sulfate (Ag_2_SO_4_) catalyst (open reflux method). Generally, mercuric sulfate (HgSO_4_) is also added to the reaction mixture to avoid interference from chloride ions. After cooling, the solution is titrated with a ferrous ammonium sulfate ((NH_4_)_2_Fe(SO_4_)_2_) solution to determine the residual dichromate, using ferroin ([Fe(C_12_H_8_N_2_)_3_]SO_4_) indicator, and compared to the value measured for the untreated sample to determine the concentration of dichromate consumed for oxidation [3]. This standard method has a few disadvantages that limit its application, such as a long reflux time, expensive chemicals (Ag_2_SO_4_), and toxic (K_2_Cr_2_O_7_, HgSO_4_) and highly corrosive (H_2_SO_4_) reagents. Nevertheless, this classic technique seems to be one of the frequently used COD measuring procedures in practice [2]. In a modified version of the method, the digestion takes place in a closed system, and spectrophotometry is used for analysis (the sealed-tube method). This method applies the smaller sample volume of 2 mL. Over the last 30–40 years, many new analytical approaches have been developed to replace persulfate solution refluxing with other digestion methods in order to avoid its drawbacks [5,6].

In laboratory investigations on selected organic compounds, the experimental COD_meas_ values are often compared with the theoretically calculated ones (ThOD) using the chemical composition and molar concentration [7]. ThOD is the stoichiometric amount of O_2_ needed to transform a C_c_H_h_Cl_cl_N_n_Na_na_O_o_P_p_S_s_ compound to CO_2_, NH_3_, H_2_PO_4_^−^, and H_2_O. The ThOD is calculated through the equation [3]:

(1) $${ThOD}_{mg/mg}=\frac{16[2c+½(h-cl-3n)+3s+5/2p+½na-o]}{M}$$

where M is the molecular mass. This equation may be used in the interactive mode through the internet. The calculation gives the theoretical COD in mg/mg units (ThOD_mg/mg_) (https://www.aropha.com/thod-calculator.html, accessed on 17 December 2023). We obtain the ThOD in the usual mg O_2_ dm^−3^ unit by multiplying the value with 1000 × M × conc; conc is the concentration in mol dm^−3^ unit. The calculation assumes that, during treatment, N transforms to NH_3_ (no nitrification).

The COD_meas_ values can differ considerably from the theoretical ones. Baker et al. [8] carried out a detailed comparison of the experimental COD values (using the measurements of Janicke et al. [9]) and calculated COD values for 565 laboratory organic chemicals, such as simple aromatics, alcohols, and aldehydes. They introduced an empirical constant, *a*, which was defined as the ratio of the measured and calculated values: *a* = COD_meas_/ThOD. The authors classified the organic compounds into six groups according to the structural characteristics. For instance, in the cases of alcohols, carbohydrates, and unsaturated carboxylic acids, the *a* values were between 0.95 and 1. However, for some classes of other compounds, e.g., hydrocarbons, several halogenated aromatic hydrocarbons, the correlation between the measured and the calculated values was very poor, with *a* less than 0.5 for some compounds. This is an important observation, because COD_meas_ analysis of the wastewater of factories releasing larger amounts of waste with compounds characterized by low *a* may give misleading results regarding the discharged organics.

The reason for the deviation of *a* from 1 is not clear; some theories have already been published in the literature. For example, some compounds may not be oxidized completely during digestion, or volatile compounds may leave the flask before complete oxidation. The low COD_meas_ values measured for some aliphatic compounds may be due to the conversion of terminal methyl groups to acetic acid or methane and not to CO_2_ [8]. Methane can be driven out of the solution before oxidation occurs.

Potassium dichromate has been proven to be carcinogenic and mutagenic, and the substance is restricted under Annex XIV of the REACH regulation (Regulation on the Registration, Evaluation, Authorization and Restriction of Chemicals) [10]. Thus, other analytical methods have been considered to replace the COD measurement to eliminate the use of potassium dichromate. The total organic carbon (TOC) content is a possible candidate: in recent years, the COD has increasingly been replaced by the TOC [11,12,13].

TOC_meas_ expresses the organic carbon content in mg C dm^−3^ units, and this amount can be determined using automatic analyzers. TOC analysis takes from 15 to 25 min and, thus, it is much faster than the classical COD measurement. The samples to be analyzed usually contain bicarbonates and carbonates (inorganic carbon (IC)). IC can be removed by acidifying the sample to a pH value of 2 or less to release IC as CO_2_, which can be measured or vented to waste. The non-purgeable organic carbon (NPOC) remaining in the liquid is then oxidized, releasing CO_2_, which is sent to the detector for measurement. Unlike COD, TOC is independent of the oxidation state of organic matter. The theoretical TOC value in mg/mg unit is calculated via the equation:

(2) $${TOC}_{mg/mg}=\frac{12\;c}{M}$$

We obtain the value in the traditional mg C dm^−3^ unit (TOC_calc_) by multiplying TOC_mg/mg_ with 1000 × M × conc.

Since the limit values of organic pollution levels in wastewater are usually described in terms of COD_meas_, efforts are being made to find a correlation between the two parameters [13]. To replace COD_meas_ with TOC_meas_, the relationship between the two quantities, i.e., the COD_meas_/TOC_meas_ ratio should be well established. In many papers, we find correlation analysis of COD_meas_ and TOC_meas_ parameters in the cases of industrial or municipal wastewater (e.g., [1,13]). The reported ratios vary in a relatively large range depending on the source of the wastewater. In the chemical industry, the COD_meas_/TOC_meas_ ratios are mostly in the range of 2.5–3.5 [1].

Here, we publish (*a*=) COD_meas_/ThOD and COD_meas_/TOC_meas_ values for a large number of individual water contaminants, including simple molecules that may serve as models for more complicated chemicals, and also for more complex molecules that are often detected in wastewater: drugs, pesticides, etc. For most of these compounds, no data are available in the literature. In the evaluation of the COD_meas_/ThOD and COD_meas_/TOC_meas_ values, we also use data taken from our previous works, determined using the same technique (described in Section 4, Materials and Methods) as in the present work. In the experimental work, a solute concentration of 0.1–0.3 mmol dm^−3^ was used in most cases. In the γ-radiolysis of three antibiotics, piperacillin, doxycycline, and erythromycin, we also investigate the changes in the COD_meas_/TOC_meas_ ratio during degradation. With these investigations, we want to answer the question of whether, in studies related to advanced oxidation processes (AOPs), the COD→TOC replacement is applicable or not.

## 2. Results

### 2.1. Data Collections, Reliability, and General Observations

Column 1 of Table 1 shows the names and chemical formulae of chemicals whose parameters are discussed here; we collected the chemical structures in the Supplementary Material. Columns 2 and 3 contain the ThOD_mg/mg_ and TOC_mg/mg_ values calculated using Equations (1) and (2). Column 4 shows the concentrations for each compound used during the measurement of the COD_meas_ and TOC_meas_ values. In the next two columns, the calculated (*a*=) COD_meas_/ThOD and COD_meas_/TOC_meas_ ratios are presented. The table contains data taken from our previous works and from other authors’ publications. In the rows, first, we show the results for some general laboratory chemicals and, then, for different classes of harmful chemical compounds. The ThOD_mg/mg_ values are concentration-independent; they are highly characteristic of individual compounds because they vary theoretically in a wide range, from methane (3.99) to oxalic acid (0.18). For saturated hydrocarbons, higher-molecular-mass alcohols, and oxo compounds (e.g., cyclohexanone), these values are around 2.5–3.5 mg/mg, while for the highly oxidized molecules, such as maleic and fumaric acids, gallic acid, and acetovanillone, the values are close to 1. The ThOD_mg/mg_ value can also be low for compounds containing several oxygen atoms or oxidizable heteroatoms (e.g., S). The theoretical TOC_mg/mg_ value of oxalic acid is 0.27; for methane, 0.75 is calculated. The theoretical ThOD_mg/mg_/TOC_mg/mg_ ratios change between 0.67 (oxalic acid) and 5.33 (methane) [1].

According to our observations, the experimental TOC values (TOC_meas_) are very close to the calculated ones (TOC_calc_): the difference between the two values is usually less than ±4%. This provided an opportunity the check the reliability of the measurements. The agreement of TOC_meas_ and TOC_calc_ values gives proof that the disagreement observed in some cases between COD_meas_ and ThOD, when both measurements were obtained from the same sample solution, is not due to any sample preparation issues or inorganic or organic impurities in the sample, but the disagreement does exist.

Table 1 shows the data measured at different concentrations for certain compounds tested in our laboratory (e.g., phenol, 2,6-dichloroaniline, *p*-aminophenol, phenolate, paracetamol); the results reasonably agree in most cases. For tetracycline, salicylic acid, and maleic acid, our COD_meas_/ThOD ratios also agree with the values calculated based on the results published in the literature. However, for chlorobenzene, there is a large difference between the value published by Baker et al. [8] and the value calculated based on the measurements of Albarran and Mendoza [18]: 0.58 and 0.25, respectively. Column 6 of Table 1 contains the ratios of the experimentally obtained COD and TOC values, COD_meas_/TOC_meas_. These data vary within a relatively narrow range; most ratios are between 2.0 and 3.0.

### 2.2. Groups of Organic Molecules

In Table 1, under the heading of laboratory chemicals, we list simple chemical compounds that can serve as starting materials for the synthesis of more complex molecules for pharmaceuticals, plant protection, paints, etc. The calculated ThOD_mg/mg_ values strongly depend on the oxidation state of the molecule and on the oxidizable heteroatoms. The smallest values, around 1, were calculated for ethylene glycol, maleic and fumaric acids, 2,4-dichlorophenol, and 2,6-dichloroaniline. Cyclohexanone, cresols, and benzamine had the highest values, around 2.5. Most of these compounds can be measured well using the dichromate/titration method; the ratios of measured and calculated COD values are around 1. A notable exception is cyclohexanone.

**Figure 1:**
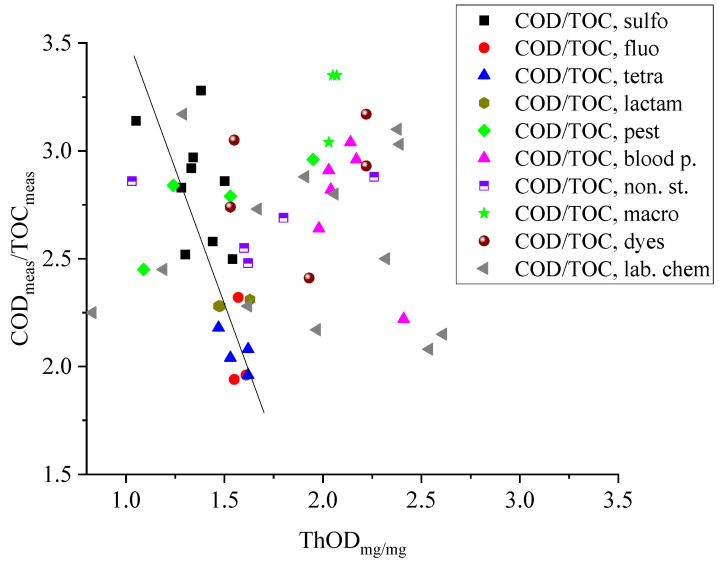
Relationship between the ThOD_mg/mg_ values and the COD_meas_/TOC_meas_ ratios.

Sulfonamide antibiotics, which prevent the multiplication of bacteria, are usually applied in combination with trimethoprim [20]. The COD_meas_/ThOD values for sulfonamides are in the 0.82–1.05 range, and the COD_meas_/TOC_meas_ ratios are between 2.52 and 3.28. As Figure 1 shows, there is some correlation between the COD_meas_/TOC_meas_ and ThOD_mg/mg_ for this group: the ratio decreases with the increasing COD values. We measured the COD and TOC values of trimethoprim at 0.1 and 0.3 mmol dm^−3^ concentrations. The COD_meas_/ThOD and COD_meas_/TOC_meas_ values at the two concentrations are different: 0.86 and 1.02, respectively, and 2.24 and 2.83, respectively. 

The broad-spectrum fluoroquinolone bactericides in Table 1, ciprofloxacin, norfloxacin, and ofloxacin share a 4-quinolone bicyclic core structure; several substituents are attached to this core [21,22]. The COD_meas_/ThOD values for fluoroquinolones vary between 0.77 and 1.03; the smallest value was measured for ofloxacin. In this molecule, the oxazine ring attached to the central core is fragile and may decompose before reaching the digestion temperature of 150 °C. As a result, low-boiling fragments can leave the system without oxidation. The average COD_meas_/TOC_meas_ ratio for fluoroquinolones is low, at 2.07.

The COD_meas_/ThOD ratios for the tetracycline antibiotics are low; according to our measurements, they are in the 0.77–0.96 range [23]. The value calculated for tetracycline, using the data of Belkheiri et al. [24] is 0.81, lower than our value, 0.96. The tetracyclines in Table 1 each have three methyl groups. COD_meas_/ThOD ratios lower than 1 are consistent with the methyl group theory mentioned in the Introduction, although these molecules are considered thermally very stable [36]. Their COD_meas_/TOC_meas_ ratios are also low; the average is 2.07.

The four β-lactam antibiotics in Table 1, amoxicillin, oxacillin, cloxacillin, and piperacillin, have similar chemical structures [25]; their ThOD_mg/mg_ values fall in a narrow range between 1.47 and 1.63. Since their COD_meas_/ThOD values are also close to each other (0.79–0.96), the COD_meas_/TOC_meas_ ratios are also in a narrow range, with an average of 2.40.

The macrolides in Table 1, erythromycin, azithromycin, and clarithromycin, contain a large number of carbon (37–38) and hydrogen (67–72) atoms, 1 or 2 nitrogen atoms, and 12–13 oxygen atoms. Due to the similar structure, the ThOD_mg/mg_ values are in a narrow range, between 2.03 and 2.07. The COD_meas_/TOC_meas_ ratios are high; they are in the 3.03–3.35 range. The ratios of the measured and calculated COD values are close to 1. These molecules have several methyl groups, but their COD-reducing effect is not observed.

The blood pressure regulators in Table 1 (atenolol, nadolol, propranolol, metoprolol tartrate, labetalol, and acebutolol) contain an oxypropanolamine unit and benzene ring(s) [26,27]. In labetalol, there are two separate rings; in propranolol, the benzene ring is replaced by a naphthalene unit. Metoprolol is mostly used as metoprolol tartrate salt (two metoprolol and one tartrate unit); this combination was used in our experiments. For these molecules, the COD_meas_/ThOD ratios are in the 0.85–0.96 range, and the COD_meas_/TOC_meas_ values are between 2.64 and 3.04. The exception is propranolol, which contains a naphthalene moiety. The COD_meas_/ThOD and COD_meas_/TOC_meas_ ratios for propranolol are low; they are 0.72 and 2.22, respectively. We assume that this behavior is related to the stability of the naphthalene unit. On the COD_meas_/TOC_meas_–ThOD_mg/mg_ plot, the data points for blood pressure regulators are far from the range of points belonging to other compounds (Figure 1).

Salicylates (salicylic acid, acetylsalicyclic acid, and 5-sulfo-salicyclic acid), ketoprofen, diclofenac, and paracetamol are listed among the non-steroidal anti-inflammatory drugs [14,28,29]. Their COD_meas_/ThOD ratios are close to 1.0, and the COD_meas_/TOC_meas_ ratios are between 2.48 and 2.88. In Figure 1, on the COD_meas_/TOC_meas_–COD plot, the points for the three salicylates are around the straight line. Ketoprofen shows “irregular” behavior; the corresponding point is in the range where we find the points of the blood pressure regulators.

The broad-spectrum antibiotic chloramphenicol was listed among the miscellaneous drugs. Due to the high number of oxygen atoms (5) and heteroatoms (4) in the molecule, it has a very low ThOD_mg/mg_ value, 0.94. This is one of the most problematic compounds for COD measurements using the dichromate method. The measured value is significantly lower that the calculated one; the COD_meas_/ThOD ratio is 0.59. In a former publication, we reported on radiolytic degradation experiments (a type of advanced oxidation process, AOP) with chloramphenicol and found that COD increased with the progress of degradation, suggesting that the products can be more easily oxidized than the starting molecule [30]. This was attributed to the “compact” structure (whatever this may mean) of the starting molecule and to the partly oxidized forms of the products. We mention that Barker et al. [8] noted that several –NO_2_ and –Cl containing molecules exhibited measured COD values lower than the theoretical value.

The antimalarial drug amodiaquine is easily oxidized in dichromate solution [31]. The COD_meas_/TOC_meas_ ratio is in the medium range, 2.55. Clofibric acid is an essential part, and, at the same time, metabolite of several anticholesteremic drugs [32]. Its COD_meas_/TOC_meas_ ratio (2.67) is similar to that of amodiaquine.

The data for fenuron, monuron, and diuron phenylurea pesticides are from our laboratory [33,34,35]. Their COD_meas_ data agree well with the ThOD. The same is true for the data for linuron, monolinuron, and buturon reported in the work of Baker et al. [8]. The average of all six COD_meas_/ThOD values is 1.02. The high ratio is surprising, since all molecules have methyl group(s), and, with the exception of fenuron chlorine, atom(s) are attached to the aromatic ring. The COD_meas_/TOC_meas_ ratios of fenuron, monuron, and diuron are very close to each other; the average is 2.86.

The six dyes in Table 1 have considerably different structures; their ThOD_mg/mg_ values cover a wide range, between 1.23 and 2.22. Despite the wide range of ThOD, the COD_meas_/TOC_meas_ ratios fall in a narrower range; they are between 2.41 and 3.17.

### 2.3. Application of COD and TOC Values in an AOP

**Figure 2:**
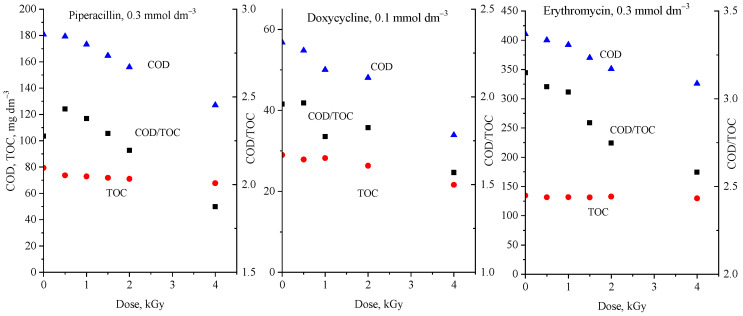
Dose dependence of the COD (blue triangle), TOC (red circle), and COD/TOC (black square) values in the γ-radiolytic degradation of piperacillin, doxycycline, and erythromycin.

Chemical oxygen demand (COD) and total organic carbon (TOC) measurements are often used in advanced oxidation processes (AOPs) to monitor the progress of degradation (e.g., [37,38]). Sometimes, both parameters are followed; sometimes, it is only one of them, which nowadays is mostly the TOC. Figure 2 shows the COD and TOC values and their ratio measured during the γ-radiolytic degradation of three antibiotics: piperacillin, doxycycline, and erythromycin. In the radiolysis of an aerated, dilute aqueous solution, the degradation of organic contaminants is mainly initiated by hydroxyl radicals (^•^OH); the amount of these radicals injected into the solution is proportional to the absorbed radiation energy (*D*, absorbed dose, unit J kg^−1^ (Gray)): [^•^OH] = 2.8 × 10^−7^ × *D* mol dm^−3^ [39]. The technology based on the application of ionizing radiation (electron beam irradiation) has already been introduced on an industrial scale, treating 10,000–30,000 m^3^ day^−1^ [40,41]. As Figure 2 shows, both the COD and TOC in all three systems decrease with the absorbed dose. However, the decrease in COD is much faster than that in TOC; their ratio constantly decreases during the process.

## 3. Discussion

The experimental COD values, COD_meas_, with some exceptions (isopropanol, vinyl acetate, cyclohexanone, 2,4-dichlorophenol, chlorobenzene, tetracyclines, propranolol, chloramphenicol, methylene blue) are close to the theoretically obtained ones (ThOD). Usually, COD_meas_ is slightly smaller than the ThOD. If the mentioned compounds are disregarded, then the average value of the ratio is COD_meas_/ThOD ± σ = 0.944 ± 0.106. The correlation suggests that the ThOD can be used as a good approximation of COD_meas_. Our investigations, similarly to other studies, could not explain the significant difference between the two values observed for a few molecules [8]. It seems that the terminal methyl groups can reduce COD_meas_ in some cases, while, in others, they have no effect on the values.

In all cases, the experimental TOC values were very close to the theoretical ones. Although, theoretically, the COD_meas_/TOC_meas_ values can vary over a very wide range, for the compounds in Table 1, the values fall in the range of 2.0–3.0, with very few exceptions. For the fluoroquinolones and the tetracyclines, the COD_meas_/TOC_meas_ ratio is low; it is around 2.0. Benzoic acid, *p*-chlorophenol, cyclohexanone, and chloramphenicol also have low ratios. The ratios are high, at around 3 or above, for isopropanol, ethylene glycol, sulfonamides, and the macrolides.

The structural dependence of the COD_meas_/TOC_meas_ ratios observed for individual compounds, similarly to in investigations with domestic and industrial wastewater [1], reflects that a single multiplication factor for the TOC to approximate the COD cannot be applied. The ratio should be determined individually and, in the case of domestic and industrial wastewater, it must be checked regularly.

In the conducted AOP investigations (γ-radiolytic degradation of antibiotics), COD decreased faster than TOC as the degradation progressed. The result shows that the investigated antibiotic molecules gradually disintegrate in many steps, forming smaller, highly oxidized fragments: first of all, small molecular mass organic acids (formic acid, acetic acid, oxalic acid, etc.) and, finally, the carbon and hydrogen atoms transform to CO_2_ and H_2_O. These small organic acids were considered “ultimate acids” as they were supposed to mineralize directly to CO_2_ [42,43]. This mechanism is supported by the gradual and strong decrease in pH during the treatment generally observed in such experiments [28]. Since the COD/TOC ratio in AOP experiments changes considerably during degradation, TOC cannot be a good substitute for COD. The change in COD during the process reflects oxidation, while the TOC characterizes the final step, the transformation of fragments to CO_2_. 

## 4. Materials and Methods

### 4.1. Chemicals

The organic compounds investigated in the experiments were mainly obtained from Sigma Aldrich (Merck, Darmstadt, Germany). Pure water used in the experiments was prepared using an Adrona B30 system (Adrona SIA, Riga, Latvia), which provides high-quality water with a conductivity of 0.055 μS cm^−1^ and a total organic carbon content <2 ppb.

### 4.2. COD_meas_ Determination

In the experiments, a Behrotest TRS 200 (Behr Labor-Technik GmbH, Düsseldorf, Germany) COD digestion system was used with K_2_Cr_2_O_7_ as oxidant, Ag_2_SO_4_ as catalyst, and HgSO_4_ to eliminate the interference of Cl^−^. Test mixtures were boiled at 150 °C for 2 h, and the remaining K_2_Cr_2_O_7_ oxidizing agent was determined via titration [3]. In order to increase the accuracy, and to remain in the recommended COD range (30–700 mg dm^−3^), in several cases, we added 30 mL solution to the digestion system in contrast to the usual 10 mL. The results are averages of three titrations.

### 4.3. TOC_meas_ Determination

For the total organic carbon (TOC_meas_) content measurements, Shimadzu (Kyoto, Japan) TOC-LCSH/CSN equipment was used. This assay is based on catalytic combustion of the organic content of samples and analysis of the formed CO_2_ using non-dispersive infrared detection.

### 4.4. γ-Radiolytic Experiments

The irradiation experiments were conducted at room temperature, in the panoramic type 1.8 PBq activity ^60^Co-γ facility of the Institute of Isotopes Co., Ltd. (Budapest, Hungary). The dose rate measured using ethanol-chlorobenzene dosimetry was 2 kGy h^−1^ [44]. The time of irradiation was varied between 0.5 and 2 h. The samples were irradiated in open air, under the conditions applied, the warming up of the samples were less than 1 °C. During irradiations, the solutions were gently bubbled with air in order to avoid oxygen depletion. In radiolytic experiments, the reactive intermediates (mainly ^•^OH radicals) are produced during irradiation as a result of the radiolysis of water. Therefore, neither catalyst nor additives are required for the AOP. 

## 5. Conclusions

The sum parameters, chemical oxygen demand (COD) and total organic carbon (TOC) are often used to characterize wastewater. The same parameters are also used in AOP investigations to follow the degradation of organic pollutants. Due to the disadvantages of COD, such as the long reflux time (2 h), expensive chemicals (Ag_2_SO_4_), and toxic reagents (K_2_Cr_2_O_7_, HgSO_4_), the COD has increasingly been replaced by the TOC. The pollution limit values in wastewater treatment are established in the COD values; therefore, efforts are being made to find the correlation between these parameters. This correlation was evaluated and discussed for ~70 laboratory chemicals, pharmaceuticals, pesticides, etc. The measured COD/TOC ratios were found to be between 2.0 and 3.0; for macrolides, they were around 3.0, and for fluoroquinolones and tetracyclines, they were around 2.0. The structure dependence suggests that the ratio should be established individually. In scientific investigations connected to degradation studies in advanced oxidation processes, we do not recommend the COD→TOC replacement. Changes in COD and TOC have different meanings: decreases in COD reflect the progress of oxidation, while in TOC, the changes show the last step, the transformation of organic carbon to CO_2_. In the case of domestic and industrial wastewater, this replacement is possible when the ratio does not change much over time, but this needs regular checking. 

## Figures and Tables

**Table 1:** Data collection for chemicals with their chemical formulae, theoretical ThOD_mg/mg_ and TOC_mg/mg_, (*a*=) COD_meas_/ThOD, and experimental COD_meas_/TOC_meas_ values.

Compound, Formula	ThOD_mg/mg_	TOC_mg/mg_	Conc. mmol dm^−3^	COD_meas_/ ThOD	COD_meas/_ TOC_meas_	Reference
Laboratory chemicals						
Isopropanol, C_3_H_8_O	2.39	0.60	0.1	0.76	3.03	This work
Ethylene glycol, C_2_H_6_O_2_	1.29	0.39	0.1	1.15	3.17	This work
Vinyl acetate, C_4_H_6_O_2_	1.67	0.56	0.1	0.76	2.73	This work
Maleic acid, C_4_H_4_O_4_	0.83	0.411	4.0	0.97		[14]
				0.99		[8]
Fumaric acid,	0.83	0.411	4.0	1.07		[14]
C_4_H_4_O_4_			0.1	1.10	2.25	This work
Cyclohexanone, C_6_H_10_O	2.61	0.734	1.0	0.63	2.15	This work
Triton X-100 C_14_H_22_O(C_2_O_4_)_n_, n = 9–10			0.1		3.25	[15]
Benzoic acid, C_7_H_6_O_2_	1.97	0.690	0.1	0.89	2.17	This work
Phenol, C_6_H_5_OH	2.38	0.765	2.0	1.01	3.15	[14]
			1.0	0.99	3.08	This work
Phenolate, C_6_H_5_O^−^	2.32	0.770	5.0	1.04	2.75	[14]
			1.0	1.02	2.25	
4-Hydroxyanisol, C_7_H_8_O_2_	2.06	0.677	0.1	1.05	2.80	This work
*o*-Cresol, CH_3_C_6_H_4_OH	2.52	0.776	1.5	0.97		[14]
*m*-Cresol, CH_3_C_6_H_4_OH	2.52	0.776	1.5	1.07		[14]
*p*-Cresol, CH_3_C_6_H_4_OH	2.52	0.776	1.5	0.92		[14]
*o*-Chlorophenol, ClC_6_H_4_OH	1.62	0.560	2.0	1.00		[14]
*m*-Chlorophenol, ClC_6_H_4_OH	1.62	0.560	2.0	1.06		[14]
*p*-Chlorophenol, ClC_6_H_4_OH	1.62	0.560	2.0	0.99		[14]
			0.3	0.67		[16]
			0.1	0.95	2.28	This work
2,4-dichlorophenol, C_6_H_4_Cl_2_O	1.18	0.442	0.1	0.72		[17]
Chlorobenzene, C_6_H_5_Cl	1.99	0.640		0.58		[8]
			0.09	0.25		[18]
*o*-Aminophenol, C_6_H_7_NO	1.91	0.660	1.0	1.04		[14]
*m*-Aminophenol, C_6_H_7_NO	1.91	0.660	1.0	1.02		[14]
*p*-Aminophenol, C_6_H_7_NO,	1.91	0.660	1.0	1.04		[14]
			1.5	0.92		
			1.0	1.00	2.88	This work
2,6-Dichloraniline, C_6_H_5_Cl_2_N	1.19	0.444	0.5	0.93	2.34	[14]
			1.0	0.89	2.50	
			0.1	1.00	2.59	This work
Benzamine, C_7_H_9_N	2.54	0.784	0.1	0.86	2.08	This work
Sulfonamides						
Sulfanilic acid, C_6_H_7_NO_3_S	1.29	0.416		1.02		[19]
Sulfamethoxazole, C_10_H_11_N_3_O_3_S	1.33	0.474	0.1	1.00	2.92	[20]
Sulfacetamide C_8_H_10_N_2_O_3_S	1.34	0.448	0.1	0.99	2.97	[20]
Sulfanilamide, C_6_H_8_N_2_O_2_S	1.30	0.418	0.1	0.82	2.52	[20]
Sulfadiazine, C_10_H_10_N_4_O_2_S	1.28	0.480	0.1	1.05	2.83	[20]
Sulfaguanidine C_7_H_10_N_4_O_2_S	1.05	0.392	0.1	1.00	3.14	[20]
Sulfathiazole, C_9_H_9_N_3_O_2_S_2_	1.38	0.423	0.1	0.98	3.28	[20]
Sulfamethazine, C_12_H_14_N_4_O_2_S	1.50	0.517	0.1	0.96	2.86	[20]
Sulfisoxazole, C_11_H_13_N_3_O_3_S	1.44	0.494	0.1	0.90	2.58	[20]
Trimethoprim, C_14_H_18_N_4_O_3_	1.54	0.579	0.1	0.86	2.24	This work
			0.3	1.02	2.83	
Fluoroquinolones						
Ciprofloxacin, C_17_H_18_FN_3_O_3_	1.61	0.616	0.1	1.03	1.96	[21]
Norfloxacin, C_16_H_18_FN_3_O_3_	1.57	0.601	0.1	0.89	2.32	[21]
Ofloxacin, C_18_H_20_FN_3_O_4_	1.55	0.598	0.1	0.77	1.94	[22]
Tetracyclines						
Tetracycline, C_22_H_24_N_2_O_8_	1.62	0.594	0.1	0.96	2.61	[23]
			0.22	0.81	2.22	[24]
Chlortetracycline, C_22_H_23_ClN_2_O_8_	1.47	0.554	0.1	0.87	2.18	[23]
Doxycycline, C_22_H_24_N_2_O_8_	1.62	0.594	0.1	0.79	1.96	This work
Oxytetracycline, C_22_H_24_N_2_O_9_	1.53	0.573	0.1	0.77	2.04	This work
β-Lactam antibiotics						
Amoxicillin, C_16_H_19_N_3_O_5_S	1.53	0.525	0.1	0.83	2.74	This work
Oxacillin, C_19_H_19_N_3_O_5_S	1.63	0.568	0.1	0.96	2.31	[25]
Cloxacillin, C_19_H_18_ClN_3_O_5_S	1.47	0.523	0.1	0.81	2.28	[23]
Piperacillin, C_23_H_27_N_5_O_7_S	1.48	0.533	0.3	0.79	2.28	This work
Macrolides						
Erythromycin, C_37_H_67_NO_13_	2.03	0.605	0.3	0.92	3.04	This work
Azithromycin, C_38_H_72_N_2_O_12_	2.07	0.609	0.1	0.98	3.35	This work
Clarithromycin, C_38_H_69_NO_13_	2.05	0.610	0.1	1.06	3.35	This work
Blood pressure regulators						
Atenolol, C_14_H_22_N_2_O_3_	1.98	0.542	0.1	0.85	2.64	[26]
Nadolol, C_17_H_27_NO_4_	2.17	0.659	0.1	0.92	2.96	[27]
Propranolol, C_16_H_21_NO	2.41	0.740	0.1	0.72	2.22	[26]
Metoprolol tartrate, C_34_H_56_N_2_O_10_	2.03	0.596	0.1	0.85	2.91	This work
Labetalol, C_19_H_24_N_2_O_3_	2.14	0.694	0.1	0.96	3.04	This work
Acebutolol, C_18_H_28_N_2_O_4_	2.04	0.642	0.3	0.95	2.99	This work
			0.1	0.88	2.64	
Non-steroidal anti-inflammatory drugs						
Salicylic acid, C_7_H_6_O_3_	1.62	0.608	1.0	0.97	2.48	[28]
			0.1	1.02		[29]
Acetylsalicylic acid, C_9_H_8_O_4_	1.60	0.600	1.0	0.97	2.55	[28]
			1.5	0.97	2.54	
5-Sulfo-salicylic acid, C_7_H_6_O_6_S	1.03	0.385	1.0	0.89	2.86	[28]
Ketoprofen, C_16_H_14_O_3_	2.26	0.755	0.4	0.97	2.88	[14]
Diclofenac, C_14_H_11_Cl_2_NO_2_	1.57	0.567	0.5	0.99		[14]
Paracetamol, C_8_H_9_NO_2_	1.80	0.635	1	0.93		[14]
				0.95	2.69	This work
Miscellaneous drugs						
Chloramphenicol, C_11_H_12_Cl_2_N_2_O_5_	0.94	0.409	1.0	0.59	1.41	[30]
Amodiaquine, C_20_H_22_ClN_3_O	2.03	0.674	0.1	1.10	2.55	[31]
Clofibric acid, C_10_H_11_ClO_3_	1.64	0.559	1.0	0.91	2.67	[32]
Pesticides						
Fenuron, C_9_H_12_N_2_O	1.95	0.658	0.1	1.00	2.96	[33]
Monuron, C_9_H_11_ClN_2_O	1.53	0.544	0.1	1.02	2.79	[34]
Diuron, C_9_H_10_Cl_2_N_2_O	1.24	0.463	0.1	1.05	2.84	[35]
Monolinuron, C_9_H_11_ClN_2_O_2_	1.34	0.503		1.11		[8]
Linuron, C_9_H_10_Cl_2_N_2_O_2_	1.40	0.433		1.06		[8]
Buturon, C_12_H_13_ClN_2_O	1.76	0.608		0.90		[8]
2,4-Dichlorophenoxy-acetic acid, C_8_H_6_Cl_2_O_3_	1.09	0.434	2.0	0.92	2.45	[14]
Dyes						
Acid Red 1, C_18_H_13_N_3_Na_2_O_8_S_2_	1.23	0.424	1.5	0.96		[14]
Chlorophenol red, C_19_H_12_Cl_2_O_5_S	1.55	0.539	0.1	1.06	3.05	This work
Methylene blue, C_16_H_18_N_3_S^+^	2.22	0.675	0.3	0.70	2.93	This work
Tetrazolium violet, C_23_H_17_N_4_^+^	2.22	0.790	0.3	0.98	3.17	This work
Ninhydrin, C_9_H_6_O_4_	1.53	0.606	0.1	1.03	2.74	This work
Thionine, C_12_H_10_N_3_S^+^	1.93	0.631	0.1	0.91	2.41	This work
Natural water contaminants						
Acetovanillone, C_9_H_10_O_3_	1.93	0.650	0.5	0.96		[14]
			0.5	1.19		
Gallic acid, C_7_H_6_O_5_	1.13	0.494	2.0	0.89		[14]

## Data Availability

The data presented in this study are available on request from the corresponding author. The data are not publicly available due to the system applied in our institute insert reason here.

## Supplementary Materials

The following supporting information can be downloaded at: https://www.mdpi.com/article/10.3390/molecules29020405/s1, Scheme S1: Chemical structures of organic molecules whose COD, TOC, and other parameters were discussed in the publication.

## References

[1] Anagnostopoulos A., Hjort M., Vaiopoulou E. Assessment of Chemical Oxygen Demand/Total Organic Carbon (COD/TOC) ratios in refinery effluents. Report: Environmental Science for European Refining, no. 16/22. Brussels, 2022. https://www.concawe.eu/publications/?_sft_topic=manufacturing-water-wastewater-emissions&_sft_publicationscategory=report.

[2] Aguilar-Torrejón J.A., Balderas-Hernández P., Roa-Morales G., Barrera-Díaz C.E., Rodríguez-Torres I., Torres-Blancas T. (2023). Relationship, importance, and development of analytical techniques: COD, BOD, and, TOC in water—An overview through time. SN Appl. Sci..

[3] OECD (1992). Guidelines for the Testing of Chemicals Ready Biodegradability Section 3.

[4] (1998). Water Quality. Determination of the Biochemical Oxygen Demand after N Days (BOD[n]) of Water-Part 1: Dilution and Seeding Method with Allylthiourea Addition.

[5] Li J., Luo G., He L.J., Xu J., Lyu J. (2018). Analytical approaches for determining chemical oxygen demand in water bodies: A review. Crit. Rev. Anal. Chem..

[6] Wu D., Hu Y., Liu Y. (2022). A review of detection techniques for chemical oxygen demand in wastewater. Am. J. Biochem. Biotechnol..

[7] Kim Y.-C., Sasaki S., Yano K., Ikebukuro K., Hashimoto K., Karube I. (2000). Relationship between theoretical oxygen demand and photocatalytic chemical oxygen demand for specific classes of organic chemicals. Analyst.

[8] Baker J.R., Milke M.W., Michelcic J.R. (1999). Relationship between chemical and theoretical oxygen demand for specific classes of organic chemicals. Water Res..

[9] Janicke W. (1983). Chemische Oxidierbarkeit Organischer Wasserinhaltstoffe.

[10] Annex XIV of the REACH Regulation (Regulation on the Registration, Evaluation, Authorisation and Restriction of Chemicals). https://echa.europa.eu/hu/authorisation-list.

[11] Choi I.-W., Kim J.-H., Im J.-K., Park T.-J., Kim S.-Y., Son D.-H., Huh I.-A., Rhew D.-H., Yu S.-J. (2015). Application of TOC standards for managing refractory organic compounds in industrial wastewater. J. Korean Soc. Water Environ..

[12] Tian X., Zhao C., Ji X., Feng T., Liu Y., Bian D. (2019). The correlation analysis of TOC and COD_Cr_ in urban Sewage treatment. E3S Web Conf..

[13] Park J.W., Kim S.Y., Noh J.H., Bae Y.H., Lee J.W., Maeng S.K. (2022). A shift from chemical oxygen demand to total organic carbon for stringent industrial wastewater regulations: Utilization of organic matter characteristics. J. Environ. Manag..

[14] Homlok R., Takács E., Wojnárovits L. (2013). Degradation of organic molecules in advanced oxidation processes: Relation between chemical structure and degradability. Chemosphere.

[15] Rácz G., Csay T., Takács E., Wojnárovits L. (2017). Degradation of Triton X-100 surfactant/lipid regulator systems by ionizing radiation in water. J. Radioanal. Nucl. Chem..

[16] Albarrán G., Mendoza E. (2020). Radiolysis induced degradation of 1,3-dichlorobenzene and 4-chlorophenol in aqueous solution. Radiat. Phys. Chem..

[17] Albarrán G., Mendoza E. (2019). Radiolytic oxidation and degradation of 2,4-dichlorophenol in aqueous solutions. Environ. Sci. Pollut. Res..

[18] Albarrán G., Mendoza E. (2020). Radiolytic degradation of chlorobenzene in aerated and deoxygenated aqueous solutions. Environ. Sci. Pollut. Res..

[19] Kishimoto N., Okumura M. (2018). Feasibility of mercury-free chemical oxygen demand (COD) test with excessive addition of silver sulfate. J. Water Environ. Technol..

[20] Sági G., Csay T., Szabó L., Pátzay G., Csonka E., Takács E., Wojnárovits L. (2015). Analytical approaches to the OH radical induced degradation of sulfonamide antibiotics in dilute aqueous solutions. J. Pharm. Biomed. Anal..

[21] Tegze A., Sági G., Kovács K., Homlok R., Tóth T., Mohácsi-Farkas C., Wojnárovits L., Takács E. (2018). Degradation of fluoroquinolone antibiotics during ionizing radiation treatment and assessment of antibacterial activity, toxicity and biodegradability of the products. Radiat. Phys. Chem..

[22] Wojnárovits L., Homlok R., Kovács K., Bezsenyi A., Takács E. (2023). Ionizing radiation induced removal of ofloxacin, abatement its toxicity and antibacterial activity in various water matrices. Appl. Sci..

[23] Wojnárovits L., Wang J., Chu L., Tóth T., Kovács K., Bezsenyi A., Szabó L., Homlok R., Takács E. (2022). Matrix effect in the hydroxyl radical induced degradation of β-lactam and tetracycline type antibiotics. Radiat. Phys. Chem..

[24] Belkheiri D., Fourcade F., Geneste F., Floner D., Aït-Amar H., Amrane A. (2015). Combined process for removal of tetracycline antibiotic–Coupling pretreatment with a nickel-modified graphite felt electrode and a biological treatment. Int. Biodeter. Biodegr..

[25] Takács E., Wang J., Chu L., Tóth T., Kovács K., Bezsenyi A., Szabó L., Homlok R., Wojnárovits L. (2022). Elimination of oxacillin, its toxicity and antibacterial activity by using ionizing radiation. Chemosphere.

[26] Kovács K., Simon Á., Tóth T., Wojnárovits L. (2022). Free radical chemistry of atenolol and propranolol investigated by pulse and gamma radiolysis. Radiat. Phys. Chem..

[27] Kovács K., Tegze A., Bezsenyi A., Wojnárovits L. (2023). Hydroxyl radical induced degradation of the ß-blocker Nadolol and comparison with Propranolol. J. Environ. Chem. Eng..

[28] Szabó L., Tóth T., Homlok R., Rácz G., Takács E., Wojnárovits L. (2014). Hydroxyl radical induced degradation of salicylates in aerated aqueous solution. Radiat. Phys. Chem..

[29] Albarrán G., Mendoza E. (2018). Ionizing radiation induced degradation of salicylic acid. Radiat. Phys. Chem..

[30] Csay T., Rácz G., Takács E., Wojnárovits L. (2012). Radiation induced degradation of pharmaceutical residues in water: Chloramphenicol. Radiat. Phys. Chem..

[31] Kovács K., Simon Á., Balogh G.T., Tóth T., Wojnárovits L. (2020). High energy ionizing radiation induced degradation of amodiaquine in dilute aqueous solution: Radical reactions and kinetics. Free Radic. Res..

[32] Csay T., Rácz G., Salik Á., Takács E., Wojnárovits L. (2014). Reactions of clofibric acid with oxidative and reductive radicals–products, mechanisms, efficiency and toxic effects. Radiat. Phys. Chem..

[33] Kovács K., Mile V., Csay T., Takács E., Wojnárovits L. (2014). Hydroxyl radical-induced degradation of fenuron in pulse and gamma radiolysis: Kinetics and product analysis. Environ. Sci. Pollut. Res..

[34] Kovács K., He S., Mile V., Földes T., Pápai I., Takács E., Wojnárovits L. (2016). Ionizing radiation induced degradation of monuron in dilute aqueous solution. Radiat. Phys. Chem..

[35] Kovács K., He S., Mile V., Csay T., Takács E., Wojnárovits L. (2015). Ionizing radiation induced degradation of diuron in dilute aqueous solution. Chem. Cent. J..

[36] Hassani M., Lázaro R., Pérez C., Condón S., Pagán R. (2008). Thermostability of oxytetracycline, tetracycline, and doxycycline at ultrahigh temperatures. J. Agric. Food Chem..

[37] Hayati F., Khodabakhshi R.K., Isari A.A., Sina Moradi S., Kakavandi B. (2020). LED-assisted sonocatalysis of sulfathiazole and pharmaceutical wastewater using N,Fe co-doped TiO_2_@SWCNT: Optimization, performance and reaction mechanism studies. J. Water Proc. Eng..

[38] Kakavandi B., Ahmadi M. (2019). Efficient treatment of saline recalcitrant petrochemical wastewater using heterogeneous UV-assisted sono-Fenton process. Ultrason. Sonochem..

[39] Buxton G.V., Spotheim-Maurizot M., Mostafavi M., Douki T., Belloni J. (2008). An overview of the radiation chemistry of liquids. Radiation Chemistry: From Basics to Applications in Material and Life Sciences.

[40] IAEA (2007). Radiation Processing, Environmental Applications.

[41] CGN (2020). 20IAEA. https://www.iaea.org/newscenter/news/started-with-iaea-support-chinas-electron-beamindustry-opens-worlds-largest-wastewater-treatment-facility.

[42] Moreira F.C., Garcia-Segura S., Boaventura R.A.R., Brillas E., Vilar V.J.P. (2014). Degradation of the antibiotic trimethoprim by electrochemical advanced oxidation processes using a carbon-PTFE air-diffusion cathode and a boron-doped diamond or platinum anode. Appl. Catal. B.

[43] Giannakis S., Gamarra Vives F.A., Grandjean D., Magnet A., De Alencastro L.F., Pulgarin C. (2015). Effect of advanced oxidation processes on the micropollutants and the effluent organic matter contained in municipal wastewater previously treated by three different secondary methods. Water Res..

[44] (2017). Practice for Use of the Ethanol-Chlorobenzene Dosimetry System.

