# Supplementary material for: Wastewater Characterization: Chemical Oxygen Demand or Total Organic Carbon Content Measurement?
Source: Molecules. 2024 Jan 14;29(2):405. doi: 10.3390/molecules29020405 (PMC10818771; doi:10.3390/molecules29020405)
Supplement: Supplementary file 1 [file molecules-29-00405-s001.zip › molecules-2806168-supplementary.pdf]

Wastewater characterization: chemical oxygen demand or total organic carbon content measurement?

László Wojnárovits [1], Renáta Homlok [1], Krisztina, Kovács [1] Anna Tegze [1], Ezsébet Takács [1],*

[1] HUN-REN Centre for Energy Research, 1121-Budapest, Konkoly-Thege Miklós út 29-33. Hungary

* Correspondence: takacs.erzsebet@ek.hun-ren.hu

Scheme S1. Chemical structures of organic molecules whose COD, TOC, etc., parameters were discussed in the publication.

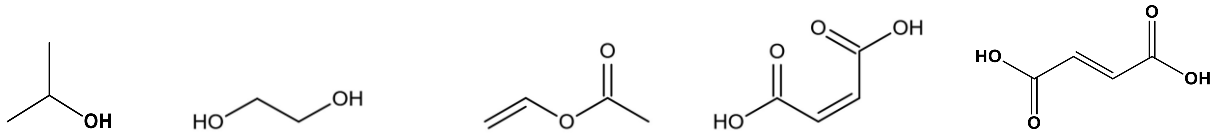

Isopropanol Ethylene glycol Vinyl acetate Maleic acid Fumaric acid

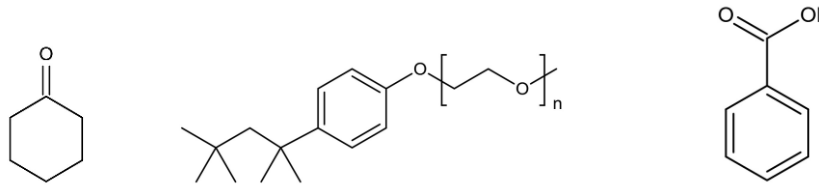

Cyclohexanone Triton X-100 Benzoic acid

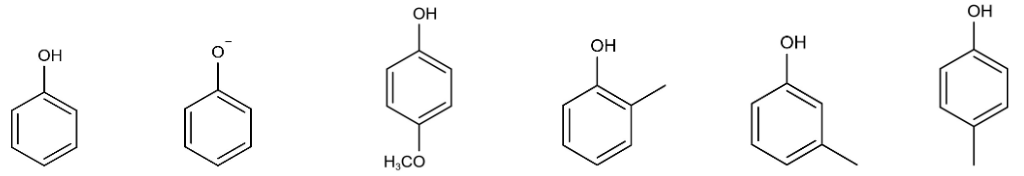

Phenol Phenolate 4-Hydroxyanisol *o*-Cresol *m*-Cresol *p*-Cresol

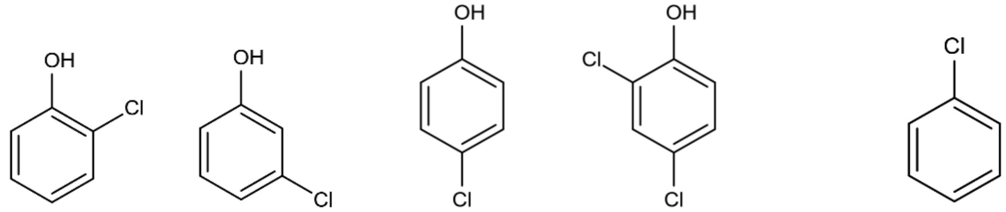

o-Chlorophenol *m*-Chlorophenol *p*-Chlorophenol 2,4-Dichlorophenol Chlorobenzene

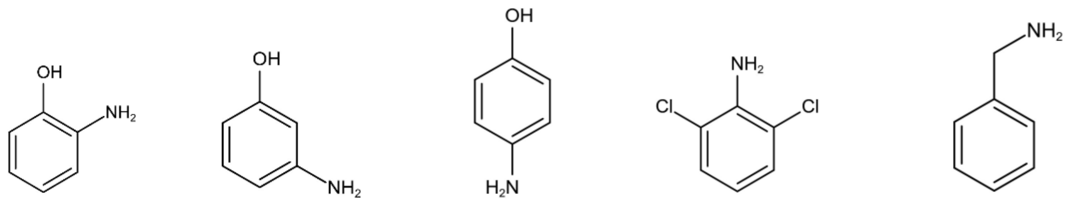

o-Aminophenol *m*-Aminophenol *p*-Aminophenol 2,6-Dichloroaniline Benzamine

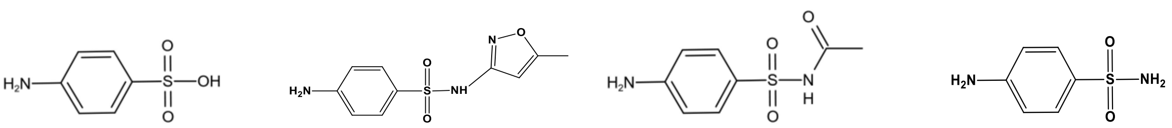

Sulfanilic acid Sulfamethoxazole Sulfacetamide Sulfanilamide

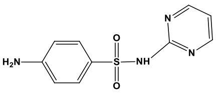

Sulfadiazine

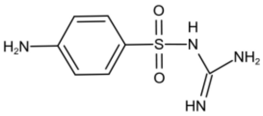

Sulfaguanidine

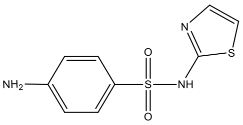

Sulfathiazole

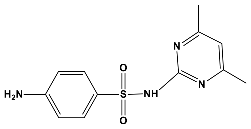

Sulfamethazine

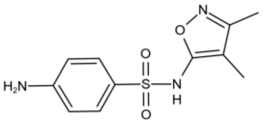

Sulfisoxazole

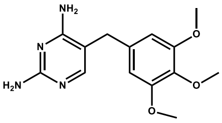

Trimethoprim

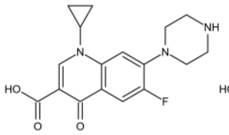

Ciprofloxacin

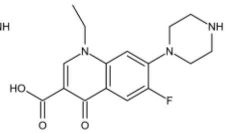

Norfloxacin

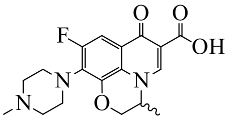

Ofloxacin

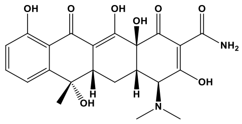

Tetracycline

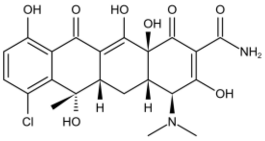

Chlorotetracycline

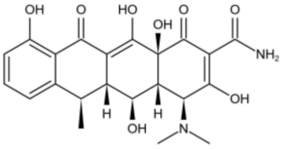

Doxycycline

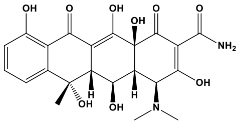

Oxytetracycline

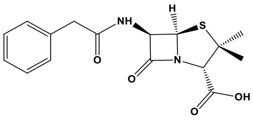

Amoxicillin

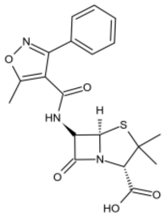

Oxacillin

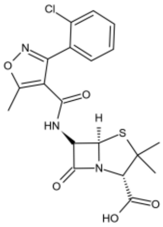

Cloxacillin

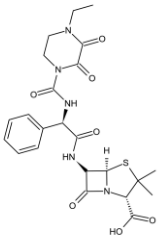

Piperacillin

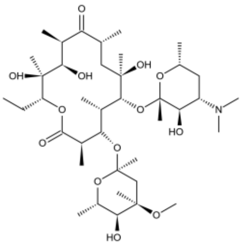

Erythromycin

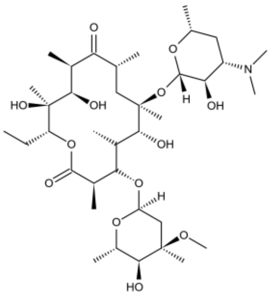

Azithromycin

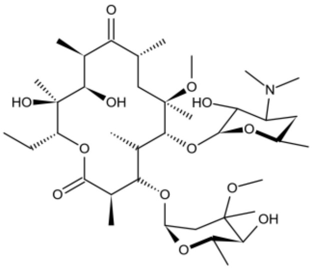

Clarithromycin

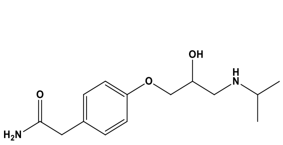

Atenolol

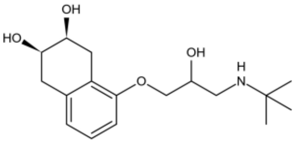

Nadolol

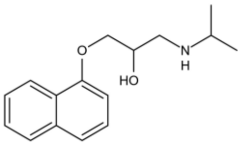

Propranolol

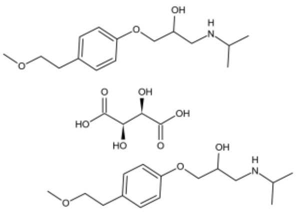

Metoprolol tartrate

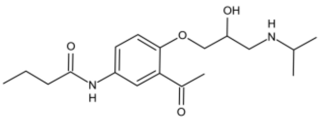

Labetalol

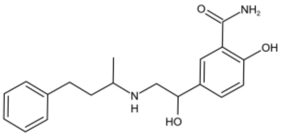

Acebutolol

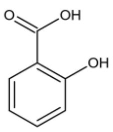
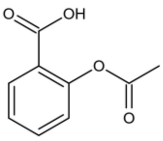
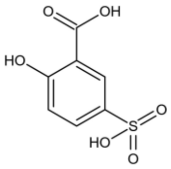
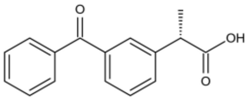
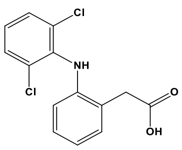

Salicylic acid Acetylsalicilyc acid 5-Sulfosalicyclic acid Ketoprofen Diclofenac

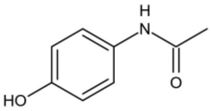
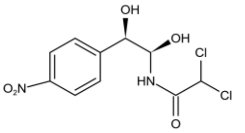
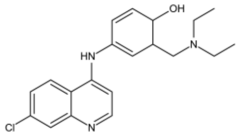
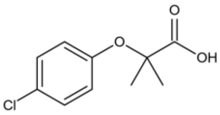

Paracetamol Chloramphenicol Aminodiaquine Clofibric acid

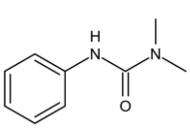
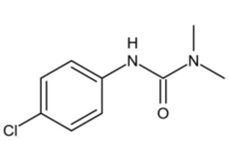
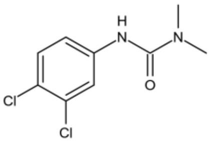
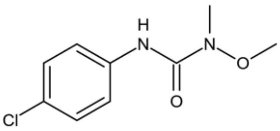

Fenuron Monuron Diuron Monolinuron

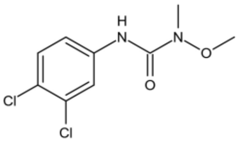
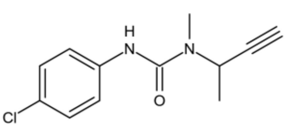
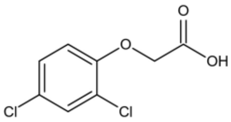

Linuron Butoron 2,4-D

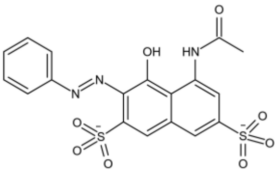
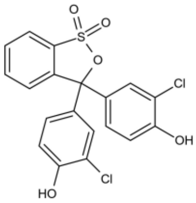
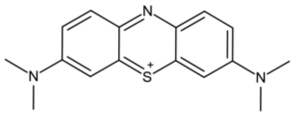

Acid red 1 Chlorophenol red Methylene blue

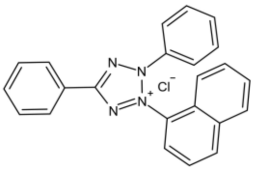
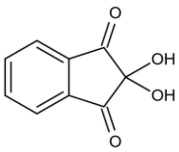
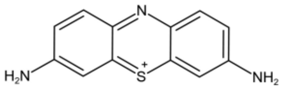
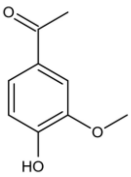
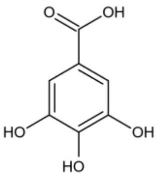

Tetrazolim violet Ninhydrin Thionine Acetovanillone Gallic acid
